# Editorial: Advances in rehabilitation for motor symptoms in neurodegenerative disease

**DOI:** 10.3389/fnhum.2023.1107061

**Published:** 2023-02-16

**Authors:** Hiroshi Kataoka, Akiyoshi Matsugi, Yasutaka Nikaido, Naoya Hasegawa, Tsubasa Kawasaki, Yohei Okada

**Affiliations:** ^1^Department of Neurology, Nara Medical University, Kashihara, Nara, Japan; ^2^Faculty of Rehabilitation, Shijonawate Gakuen University, Osaka, Japan; ^3^Clinical Department of Rehabilitation, Osaka Medical and Pharmaceutical University Hospital, Osaka, Japan; ^4^Graduate School of Health Sciences, Hokkaido University, Hokkaido, Japan; ^5^Department of Physical Therapy, School of Health, Institute of Sports Medicine and Science, Tokyo International University, Saitama, Japan; ^6^Graduate School of Health Sciences, Kio University, Nara, Japan

**Keywords:** Parkinson, rehabilitation, motor activity, melatonin, wearing-off

This Research Topic highlights seven studies that present scientific perspectives on objective assessments, neural mechanisms, and rehabilitation interventions for motor symptoms in neurodegenerative diseases.

Freezing of gait (FOG) is a common symptom of Parkinson's disease (PD) and related disorders. FOG is defined as “a brief episodic absence or marked reduction in the forward progression of the feet despite the intention to walk” (Nutt et al., 2011). Accurate assessments of the temporal characteristics and phenotypes (i.e., shuffling, trembling, and complete akinesia) of FOG are important for the rehabilitation of patients with PD. Videos are commonly used to assess FOG severity in patients with PD. Kondo et al. reported that the measurement accuracy of video-based FOG assessments during the Timed Up and Go Test is high for the temporal characteristics of FOG but low for the phenotypes of FOG. This finding provides useful information for the objective assessment of FOG in patients with PD.

Postural instability in response to backward disturbances is prominent in patients with PD (Horak et al., 2005), which increases the risk of backward falls. Compensatory step training, particularly against backward disturbances, is essential for reducing the risk of falls due to postural instability. Hasegawa et al. reported that repeated step training against backward disturbance, with or without anticipation of disturbance, improved step length and margin of stability, which are considered objective measures of dynamic stability in healthy subjects. This finding may provide a basis for the rehabilitation of backward instability in patients with PD.

Bradykinesia is a common motor symptom in PD. Finger tapping is commonly used to assess bradykinesia in patients with PD (Picillo et al., 2015). To develop rehabilitative interventions, a better understanding of the underlying neural mechanisms of PD symptoms is required. Li et al. attempted to determine a reliable mechanism of cortical activation during finger tapping under cue-priming conditions in patients with PD through a meta-analysis of 15 studies. They found that patients with PD showed specific activation under cue-priming conditions, indicating that cue-priming increases the executive control of finger movements in patients with PD. These findings suggest that cue-priming is beneficial for rehabilitating patients with PD.

Music-based interventions such as singing are expected to improve motor and non-motor symptoms in patients with PD (Butala et al., 2022). Group therapeutic singing demonstrated a trend toward greater improvement of motor symptoms and sadness and a reduction in cortisol levels as a physiological measure of stress in patients with PD. Further studies on the potential mechanisms of singing as an intervention for motor symptoms in PD will provide greater insights into the therapeutic use of music in the rehabilitation of patients with PD.

Transcranial direct current stimulation (tDCS) is expected to be a beneficial rehabilitative intervention for the motor symptoms of neurodegenerative diseases, such as PD (Sadler et al., 2022) and spinocerebellar degeneration (Maas et al., 2022). Because many situations in daily life require motor control for decision-making, effective treatments are needed. Yamamoto et al. developed a motor control task that required decision-making and studied the effects of anodal tDCS on the left dorsolateral prefrontal cortex (DLPFC) in healthy subjects *via* a randomized, sham-controlled trial. They found that anodal tDCS of the left DLPFC improved the performance of subjects in the task. This finding may provide a basis for developing rehabilitative interventions for motor performances that requires cognitive functions such as decision-making.

Charcot-Marie-Tooth disease (CMT) is a progressive sensory and motor neuropathy of the peripheral nerves that causes severe motor deficits in the lower limbs. Rambelli et al. conducted a scoping review of the clinical scale of foot deviations in CMT. This study highlighted six scales as important: the “Foot Posture Index” (FPI), “Foot Function Index,” “Maryland Foot Score,” “American Orthopedic Foot & Ankle Society's Hindfoot Evaluation Scale,” “Foot Health Status Questionnaire,” and “Wicart–Seringe grade.” Furthermore, the Rasch analysis suggested the adoption of the six-item version of the FPI for foot assessment in CMT disease. These findings are critical for the neurorehabilitation assessment of gait disturbances in individuals with CMT and are worthy of consideration in future research and clinical practice.

In patients with neurodegenerative diseases, devising treatment for improving hand motor control for tasks such as assembly is an unmet medical need. Degenerative changes in the local brain regions or the entire brain affect functional neural connectivity and networks, resulting in motor deficits. Taniguchi et al. reported that functional magnetic resonance imaging can identify disparities in task-related functional connectivity between trained and untrained participants using a tool for novel tasks. Their findings lend credence to the hypothesis that assembly-trained workers exhibit stronger connectivity between the cerebellum and tool-specific motor areas, whereas untrained individuals demonstrate greater involvement of sensorimotor networks during tool-usage tasks. This discovery is of paramount importance, as it furthers our understanding and paves the way for the development of rehabilitative strategies to train individuals with neurodegenerative diseases in novel motor tasks.

The findings from these studies could contribute to the advancement of the neural mechanisms involved, objective assessment tools, and appropriate rehabilitation interventions to treat the motor symptoms of neurodegenerative diseases. Various rehabilitation interventions have been developed; however, specific assessment methods have not yet been established. Particularly in patients with PD, various motor symptoms have been reported; however, the objective assessment or neural mechanisms of these motor symptoms remain unclear. Motor disabilities in PD develop according to the severity and progression of the disease. PD is a common progressive neurodegenerative disease in aging individuals caused by a reduction in dopamine production in brain cells. The cardinal symptoms of PD include tremors, rigidity, and bradykinesia, which respond to levodopa administration. Oral levodopa is converted to dopamine in the brain, increasing endogenous dopamine levels; consequently, patients with PD are able to effectively control their motor symptoms and maintain a satisfactory quality of life. However, the plasma half-life of levodopa is short, which leads to alternating high and low dopamine levels in the brain. The progressive degeneration of nigrostriatal dopaminergic terminals results in a loss of dopamine storage capacity, and the duration of the therapeutic effect of levodopa starts to shorten within a few years after diagnosis. A clear motor fluctuation in levodopa benefits is observed immediately after administration (ON phase) and its disappearance (OFF phase). This phenomenon is known as “wearing-off,” and ~40% of patients with PD experience it within 5 years of their diagnosis. As the disease progresses, the levodopa benefits gradually decrease because of the occurrence of “wearing-off,” which is defined as a predictable recurrence of PD symptoms that appear before the next scheduled dose and are relieved after the administration of antiparkinsonian medications (Stacy et al., 2006). Advanced PD is associated with the wearing-off phenomenon, and the severity of motor disabilities increases during the off-state.

Stegemöller et al. investigated the effects of therapeutic singing on the motor symptoms of patients with PD. The primary outcome measure was clinical motor symptoms assessed using the Unified Parkinson's Disease Rating Scale (UPDRS) Part III scores from video recordings of the patients immediately before and after therapeutic singing. The intervention included two parts: a greeting song and a series of vocal exercises (including diaphragmatic breathing), lip buzzing, glissandos, and articulation exercises. A recent systematic review reported that although the results were not always statistically significant because of the small sample sizes, 12 studies supported the beneficial effect of singing interventions on motor symptoms, with only one study finding no evidence of a benefit (Barnish and Barran, 2020). The results of Stegemöller et al.'s study demonstrated a trend toward a decrease in motor UPDRS scores; however, this was not statistically significant. The wearing-off phenomenon in PD is well-known, and motor symptoms fluctuate within the course of a day. This study did not mention the presence of wearing-off during the motor score evaluation or singing intervention. In this study, patients with PD were old (74.29 ± 1.70, maximum 85 years), and the disease duration was relatively longer (7.5 ± 1.15, maximum 17 years). The older the patient with PD, the shorter the time from onset to wearing-off (Yoritaka et al., 2020).

Li et al. attempted to reliably determine cortical activity using a finger-tapping task through a meta-analysis of 15 studies. Many subsequent neuroimaging studies have discovered changes in brain activation in various regions, such as the primary motor cortex, supplementary motor area, parietal lobe, ventrolateral thalamic nucleus, and inferior frontal gyrus, during the finger-tapping task in patients with PD. However, the distribution of activated areas and the direction of activation changes reported in existing studies have been inconsistent. Li et al. found significant activation in seven regions, including the precentral gyrus, superior parietal lobe, cerebellum, and basal ganglia. It is unclear whether the finger-tapping task was performed during the on- or off-state. UPDRS scores in the on- and off-states were documented in 1 of the 15 studies selected, and the difference in the score between the states was large (31.3 vs. 18.9). On functional magnetic resonance imaging, functional connectivity differs between deep brain stimulation on- and off-states of motor connectivity (Kahan et al., 2019). The authors commented that the difficulty in determining clear brain activation patterns may have been caused by inconsistent priming conditions during the finger-tapping task.

Kondo et al. retrospectively investigated FOG, a common symptom in the late stages of PD, by calculating the measurement accuracy of the video-recorded timed up and go walking task. The FOG duration and percentage showed good measurement accuracy for both intra- and inter-rater reliabilities. However, FOG phenotypes (shuffling, trembling, and complete akinesia) showed poor measurement accuracy for inter-rater reliability. Patients with PD had Hoehn–Yahr stages 3 or 4 and frequently exhibited wearing-off. In the off-state, shuffling, trembling, and complete akinesia were common. If all patients underwent a Timed Up and Go test during the off-state, the FOG phenotypes could achieve good measurement accuracy.

What is an objective evaluation method for PD that considers its effect on fluctuating motor disabilities?

Motor disabilities in PD develop according to disease progression and severity. In particular, advanced PD has motor complications such as wearing-off phenomena or dyskinesia, and the severity of motor disability during the off-state is increasing. Most rehabilitation studies, particularly those on advanced PD, have measured motor activity during the on-state, and most evaluations have not included off-state motor activity. Devices such as accelerometers, video-based measurements, and questionnaires were used to assess motor activity. Actigraphy can evaluate physical activity for several weeks, and actigraph measurements transformed to a cosine curve have been widely used (Baird et al., 2012). Activity data can be fitted to protean curves, ranging from a simple cosine curve to a curve that resembles a square wave, using sigmoidally converted cosine curves and the arctangent function (Marler et al., 2006). The following diurnal physical activity parameters were evaluated: amplitude, the difference in activity from peak to trough; mesor, the mean activity level; and acrophase, the time of day of peak activity. These parameters can be used to assess the effects of rehabilitation on fluctuating motor activities. Accelerometers provide a large amount of physical activity data. Video-based evaluations or questionnaires cannot precisely examine fluctuating motor activity. Therefore, a simple marker, such as a blood sample, is necessary to assess fluctuating motor disabilities.

The daily rest-activity rhythms in PD have shown that the amplitude of physical activity is reduced and the acrophase of the activity rhythms differs as the stages develop (Obayashi et al., 2021). Moreover, non-motor symptoms, including fatigue and sleep disturbance, can significantly affect motor activity. Sleep disturbances are frequent in patients with PD, and better sleep can reduce motor disability, especially during the early morning off-state (Kataoka et al., 2020). “Sleep benefit” has been known to increase motor activity. Sleep is regulated by circadian rhythms, which may be associated with rest-activity rhythms. Melatonin released from pinealocytes may inhibit the suprachiasmatic nuclei (SCN) and influence circadian rhythms (Pandi-Perumal et al., 2008). Diurnal variations in melatonin levels are closely linked to sleep (Pandi-Perumal et al., 2008). Melatonin plays an important role in several aspects of the circadian rhythm. In patients with PD, the serum melatonin level is lower than in non-PD subjects, especially in PD patients with excessive daytime sleepiness (Videnovic et al., 2014). A significant correlation between serum melatonin levels and overall PD severity has been reported using the Hoehn and Yahr scale (Lin et al., 2014). Blight therapy also improves daily physical activity and sleep quality (Videnovic et al., 2017). Melatonin administration ameliorates poor sleep quality (Medeiros et al., 2007; Hadoush et al., 2020) and increases motor activity in patients with PD. Hypothetically, serum melatonin may be a potential biomarker for the effect of rehabilitation on fluctuating motor disability, which is a unique feature of PD.

What are alternative treatment options to singing therapy?

Administration of melatonin in MPTP-induced experimental models of PD lowered the dosage of levodopa required to achieve striatal dopamine recovery (Naskar et al., 2015). Is melatonin a potential therapeutic agent? Melatonin and exercise-based neurorehabilitation are effective for faster recovery after spinal cord injury (Hong et al., 2010). In adults with multiple sclerosis, a pilot study showed that acute nocturnal melatonin ingestion was likely to enhance postural balance and physical performance, including the Timed Up and Go test and Timed 25-foot walk test, by improving sleep quality and cognitive function (Jallouli et al., 2022). Previous clinical trials on melatonin administration have failed to show its efficacy in treating motor disability (Medeiros et al., 2007; Hadoush et al., 2020). However, melatonin has been reported to have potential neuroprotective effects, such as free radical scavenging *in vitro* in cellular models and *in vivo* in various experimental animal models (Borah and Mohanakumar, 2009; Romero et al., 2010); additionally, it may block the pro-apoptotic cascade at different levels and prevent neuroinflammation, leading to the prevention of dopaminergic neuronal death. Although further research is needed to determine whether melatonin directly reduces motor symptoms, the link between motor and non-motor symptoms, including sleep, is intensified in PD; based on the results of many clinical trials, melatonin improves sleep (Medeiros et al., 2007; Hadoush et al., 2020; Ma et al., 2022) and may ameliorate motor disability.

Thus, melatonin administration may be a potential adjunctive therapy for rehabilitation. The distinct relationship between fluctuating motor activity and circadian rhythm/melatonin needs to be clarified in future studies. In patients with PD, it may be better to consider fluctuating motor activity before rehabilitation for motor symptoms.

## Author contributions

HK was responsible for the overall study design. HK, AM, and YO wrote the manuscript, contributed to drafting, and critical revision of part of the submitted materials. All authors contributed to the article and approved the submitted version.
